# Pilot study of expanded carrier screening for 11 recessive diseases in China: results from 10,476 ethnically diverse couples

**DOI:** 10.1038/s41431-018-0253-9

**Published:** 2018-10-01

**Authors:** Sumin Zhao, Jiale Xiang, Chunna Fan, Asan, Xuan Shang, Xinhua Zhang, Yan Chen, Baosheng Zhu, Wangwei Cai, Shaoke Chen, Ren Cai, Xiaoling Guo, Chonglin Zhang, Yuqiu Zhou, Shuodan Huang, Yanhui Liu, Biyan Chen, Shanhuo Yan, Yajun Chen, Hongmei Ding, Fengyu Guo, Yaoshen Wang, Wenwei Zhong, Yaping Zhu, Yaling Wang, Chao Chen, Yun Li, Hui Huang, Mao Mao, Ye Yin, Jian Wang, Huanming Yang, Xiangmin Xu, Jun Sun, Zhiyu Peng

**Affiliations:** 1 Tianjin Medical Laboratory, BGI-Tianjin, BGI-Shenzhen, 300308 Tianjin, China; 2 Binhai Genomics Institute, BGI-Tianjin, BGI-Shenzhen, 300308 Tianjin, China; 3 BGI Genomics, BGI-Shenzhen, 518083 Shenzhen, China; 4 Department of Medical Genetics, School of Basic Medical Sciences, Southern Medical University, 510515 Guangzhou, Guangdong China; 5 Department of Hematology, 303rd Hospital of the People’s Liberation Army, Nanning, Guangxi China; 6 The Second Department of Pediatrics, Affiliated Hospital of Zunyi Medical College, Zunyi, Guizhou China; 7 Nation Health and Family Planning Commission Key Laboratory For Preconception and Health Birth in Western China, The First People’s Hospital of Yunnan Province, Kunming University of Science and Technology, Kunming, China; 8 Department of Biochemistry and Molecular Biology, Hainan Medical College, Haikou, Hainan China; 9 Department of Genetic and Metabolic Laboratory, Guangxi Zhuang Autonomous Region Women and Children Health Care Hospital, Nanning, Guangxi China; 10 Department of Medical Genetics, Liuzhou Municipal Maternity and Child Healthcare Hospital, Liuzhou, Guangxi China; 11 Maternity and Child Health Care Hospital of Foshan City, Foshan, Guangdong China; 12 Guilin Women and Children Health Care Hospital, Guilin, Guangxi China; 13 Department of Clinical Laboratory, Zhuhai Municipal Maternal and Child Healthcare Hospital, Zhuhai Institute of Medical Genetics, Zhuhai, Guangdong China; 14 Maternal and Child Health Hospital in Meizhou, Meizhou, Guangdong China; 15 Department of Prenatal Diagnosis Center, Dong Guan Maternal and Child Health Hospital, Dongguan, Guangdong China; 16 Baise Women and Children Care Hospital, Baise, Guangxi China; 17 Genetic Laboratory, Qinzhou Maternal and Child Health Hospital, Qingzhou, Guangxi China; 18 Women and Children’s Health Hospital of Shaoguan, Shaoguan, Guangdong China; 19 Department of Gynecology and Obstetrics, The People’s Hospital of Yunfu City, Yunfu, Guangdong China; 20 BGI Clinical Laboratories-Shenzhen, BGI-Shenzhen, 518083 Shenzhen, China; 21 James D. Watson Institute of Genome Sciences, 310058 Hangzhou, China; 22 BGI-Shenzhen, 518083 Shenzhen, China; 23 BGI-Guangzhou Medical Laboratory, BGI-Shenzhen, 510006 Guangzhou, China

**Keywords:** Genetic testing, Risk factors

## Abstract

Expanded carrier screening (ECS) has been demonstrated to increase the detection rate of carriers compared with traditional tests. The aim of this study was to assess the potential value of ECS for clinical application in Southern China, a region with high prevalence of thalassemia and with diverse ethnic groups, and to provide a reference for future implementations in areas with similar population characteristics. A total of 10,476 prenatal/preconception couples from 34 self-reported ethnic groups were simultaneously tested and analyzed anonymously for 11 Mendelian disorders using targeted next-generation sequencing. Overall, 27.49% of individuals without self-reported family history of disorders were found to be carriers of at least 1 of the 11 conditions, and the carrier frequency varied greatly between ethnic groups, ranging from 4.15% to 81.35%. Furthermore, 255 couples (2.43%) were identified as carrier couples at an elevated risk having an affected baby, sixty-five of which would not have been identified through the existing screening strategy, which only detects thalassemia. The modeled risk of fetuses being affected by any of the selected disorders was 531 per 100,000 (95% CI, 497–567 per 100,000). Our data demonstrate the feasibility of ECS, and provide evidence that ECS is a promising alternative to traditional one-condition screening strategies. The lessons learned from this experience should be applicable for other countries or regions with diverse ethnic groups.

## Introduction

Prenatal/preconception carrier screening, which aims to identify individuals or couples at risk of passing on recessive monogenic disorders to their offspring, has been accepted as an effective early intervention strategy to reduce the prevalence of disability and disease in the newborn population. A carrier screening program for Tay-Sachs disease started in 1971 [1] and has become the prototype for carrier screening of recessive disorders.

Advances in sequencing technology and decreases in cost [2] have made expanded carrier screening (ECS) feasible and affordable. In 2011, after 14 years of cumulative experience in gene-by-gene carrier screening, screening tests were first expanded to simultaneously test for 448 Mendelian recessive diseases using next-generation sequencing (NGS) technology [3]. Since then, ECS has been implemented in several populations, and the power of NGS and expanded panels increases detection rates compared with traditional tests [4–7].

It is known that the carrier frequencies of Mendelian disorders differ considerably between ethnic groups [7, 8]. Scientific guidelines announced by the American College of Obstetricians and Gynecologists (ACOG), the American College of Medical Genetics and Genomics (ACMG), and other professional institutes dictate careful vetting of demographic characteristics, such as ethnic background, during carrier screening [9–12]. Knowing the frequency of variants in the population being tested facilitates pan-ethnic testing, but this is generally unrealistic to achieve due to costs and concerns of stigmatization. Moreover, geographical mobility is continuously increasing, also in China [13], increasing the possibility of mixed marriages and the complexity of genetic backgrounds.

China is a populous country with high incidence of birth defects [14], and has 56 ethnic groups (the Han and 55 ethnic minorities) [15, 16] which typically have their own distinctive culture and language. Several studies have elucidated some of the genetic variation in these groups [17–19]. These population characteristics provide a good opportunity to explore the performance of ECS in area with diversified ethnicities.

Here, we report the first design and validation of an ECS panel covering 11 Mendelian disorders using targeted NGS in China. The testing of the ECS in 10,476 ethnically diverse couples simultaneously supports its feasibility and provides evidence that ECS is useful in a region with diverse ethnic groups. We also identified several groups at considerable risk of passing genetic variants to their offspring, indicating a potential need for expanded carrier screening.

## Materials and methods

### Study population

The samples were categorized into two groups. The first group included 2238 patients or carriers with known genetic variants and was used for the validation of the ECS panel. The second group consisted of samples from 10,527 couples without a self-identified family history of inherited disorders, who were selected from five provinces in southern China for a hemoglobinopathies research project [20]. The pre-test genetic counseling for the hemoglobinopathies project was free to access. Basic genetic knowledge, such as inheritance patterns was spread through posters in hospitals while the hemoglobinopathies research project was underway. These couples were informed that the remaining samples might be used for other research purposes after anonymization and de-identification. Informed consent was required for all individuals.

Fifty-one of these couples were excluded from further analysis because either or both of the partners’ DNA failed the sample quality control. Members of a couple underwent the test simultaneously and were required to report their ethnic background. Individuals in China identify as part of an ethnic group only if at least one of their parents belongs to the group. As this study was performed 1 year after sample collection, the testing results were not informed to individuals. The Institutional Review Board (IRB) of BGI approved the study.

### Disease selection and panel design

The selection of monogenic diseases followed the criteria recommended by the ACMG, including considerations regarding prenatal diagnosis and a validated clinical association between mutation(s) and disease severity [12]. Eleven recessive disorders were included, namely α-thalassemia (OMIM 604131); β-thalassemia (OMIM 613985); Phenylalanine hydroxylase deficiency (PAH deficiency; OMIM 261600); Wilson disease (WD; OMIM 277900); GJB2-related DFNB1 nonsyndromic hearing loss and deafness (DFNB1; OMIM 220290); Deafness, autosomal recessive 4, with enlarged vestibular aqueduct (DFNB4; OMIM 600791)/Pendred syndrome (PDS; OMIM 274600); Pompe disease (PD; OMIM 232300); Autosomal recessive polycystic kidney disease (ARPKD; OMIM 263200); Hyperphenylalaninemia, BH4-deficient, A (HPABH4A; OMIM 261640); Galactosemia (OMIM 230400); and Dystrophinopathies (Duchenne muscular dystrophy; OMIM 310200 & Becker muscular dystrophy; OMIM 300376). The severity of these diseases was classified as moderate, severe or profound [21] (Supplementary Table 1).

Twelve genes were selected for the design of a customized capture array (NimbleGen, Roche) to capture the target regions of interest, including all exons along with 60-bp flanking intronic sequences and certain well-characterized intron sequences. For thalassemia, in addition to the above-mentioned regions, known deleterious copy number variants (CNVs) listed in HbVar (http://globin.cse.psu.edu/hbvar/) outside exons and single nucleotide variants (SNVs)/insertion and deletion variants (indels) were included. Additionally, 21 autosomal SNPs and 2 genes (*PS4Y1* and *AMELY*) on the Y-chromosome were included for internal quality assurance. The total size of targeted regions was 386,605 bp.

### NGS and data analysis

Genomic DNA extraction was carried out with a MagPure Buffy Coat DNA Midi KF Kit (Magen, China) from peripheral blood. The DNA was then sequenced on a HiSeq 2000 or 4000 (Illumina, San Diego, USA) in 100-bp paired-end reads. The capture of targeted regions, enrichment, elution, and data analysis was performed according to a previously published protocol [20]. The reference genome sequence used was hg19.

Classification and interpretation of variants were performed according to the standards and guidelines recommended by ACMG and published in the literature [22–24]. The classification criteria used to support pathogenicity of the variants are listed in Supplementary Table 2. A variant database was constructed as previously described [25]. Our final database included a total of 2319 variants, including 1310 SNVs, 432 indels and 577 CNVs. Individuals were identified as carriers if they were positive either for variants in our database or for loss-of-function (LOF) variants not listed in our database that have an allele frequency lower than 0.001 in the 1000 Genomes Project (www.1000genomes.org/) and dbSNP (www.ncbi.nlm.nih.gov/SNP/). If both the male and the female were carriers of a same autosomal recessive disorder or the female was the carrier of an X-linked recessive disorder, they were identified as a carrier couple at increased risk having an affected baby.

## Results

### Validation study

We first evaluated the performance of the NGS-based screening assay. The Yan Huang genome sample [26] was utilized as reference material to assess the sequencing depth needed for detecting variants of target regions. When the sequencing depth was greater than 100-fold, the sensitivity, specificity, accuracy, positive predictive value, and negative predictive value of SNVs on the targeted regions (≥20 depth) reached 99.9% (Supplementary Figure 1). Coverage of the targeted regions (≥20 depth) was also above 99% (Supplementary Figure 2). We next tested the reproducibility of the assay and detected no inconsistent variants between intra- and inter-run replicates, giving a concordance rate of 100%. The consistency of the same regions in ≥20-fold coverage was over 99.8% (Supplementary Table 3-4).

We also simulated 100 genomes containing 1743 deleterious variants to appraise the performance of the assay. When the mean sequencing depth was ≥100-fold, the accuracy, specificity, and sensitivity of variant detection were all over 99.9% (Supplementary Table 5). Finally, we analyzed 2238 samples with 4322 known variants to validate the ECS panel (Supplementary Table 6). Overall, 4321 of the 4322 variants were detected. The undetected variant was the partial deletion (30 bp) of exon 52 in the *DMD* gene, which was removed during quality filtration due to the small size of deletion. In general, on the basis of the validation results, a mean sequencing depth of ≥100-fold was aimed for in the study of 10,476 couples.

### Population demographics

The screening population included 10,476 couples (20,952 individuals) with an average age of 29.23 years and a median age of 28 years. Approximately 96.71% (10,131/10,476) of the couples included ethnic information for both partners, with 2.68% (*n* = 281) having ethnic information for only one partner and 0.61% (*n* = 64) not identified to any ethnicity. In total, 34 self-reported Chinese ethnic groups were included. The largest population was the Han ethnicity (*n* = 14,233), comprising 67.93%, with minorities accounting for 30.12% (*n* = 6310) and 1.95% (n = 409) of individuals without a reported ethnic group (Supplementary Table 7). The top ten minority groups in the south of China were all represented in the study, namely the Zhuang, Hui, Miao, Yi, Tujia, Dong, Buyei, Yao, Bai, and Hani [16]. Fourteen ethnic groups which had over one hundred representatives in this study were included for further analysis and discussion.

### Disease carrier frequencies

Data about the variants identified in this study has been deposited in Genome Variation Map at http://bigd.big.ac.cn/gvm/getProjectDetail?project=GVM000025. The carrier frequencies of the 11 conditions are presented in Table 1. Of the 20,952 individuals screened, 27.49% (*n* = 5707) were found to be carriers of at least one condition. The most common disease was α-thalassemia, with a rate of 15.12%, extraordinarily high compared with other diseases detected in this study. This was four times greater than PAH deficiency (*n* = 753, 3.59%) and three times greater than β-thalassemia (*n* = 995, 4.75%). The carrier frequencies of five disorders are reported for the first time in the Chinese population: WD (*n* = 410, 1.96%), ARPKD (*n* = 118, 0.56%), HPABH4A (*n* = 52, 0.25%), galactosemia (*n* = 26, 0.12%), and dystrophinopathies (*n* = 13, 0.12% of females).

**Table 1:** Carrier frequencies of 11 recessive diseases in cohort of 10,476 couples

Disease	Carrier frequency		
	N	%^a^	1 in _
All	5707	27.49	4
α-thalassemia	3167	15.12	7
β-thalassemia	995	4.75	21
Phenylalanine hydroxylase deficiency	753	3.59	28
Wilson disease	410	1.96	51
DFNB1	347	1.66	60
DFNB4/PDS	333	1.59	63
Pompe disease	158	0.75	133
ARPKD	118	0.56	178
HPABH4A	52	0.25	403
Galactosemia	26	0.12	806
Dystrophinopathies^b^	13	0.12	806

*DFNB1* GJB2-related DFNB 1 nonsyndromic hearing loss and deafness, *DFNB4* deafness, autosomal recessive 4, with enlarged vestibular aqueduct, *PDS* Pendred syndrome, *ARPKD* autosomal recessive polycystic kidney disease, *HPABH4A* hyperphenylalaninemia, BH4-deficient, A

^a^Percentage of 20,952 individuals screened

^b^Carrier frequency in female

### Carrier frequencies categorized by ethnicity

The overall carrier frequencies varied substantially between ethnic groups, ranging from 4.15% of individuals of Hani ethnicity to 81.35% of individuals of Li ethnicity (Table 2). Three ethnic groups (the Dai, Zhuang, and Yao) demonstrated high positive rates for at least one disease on the ECS panel (39.82%, 35.70%, and 29.17%, respectively). The Han ethnicity, which is the largest ethnic group in China, had an overall carrier frequency of 25.28%.

**Table 2:** Carrier frequencies categorized by ethnicity

Ethnicity	Number of screened	Total carrier frequency			Carriers for one disease			Carriers for two or more diseases		
		*N*	%	1 in _	*N*	%	1 in _	*N*	%	1 in _
All	20,952	5707	27.49	3.6	5083	24.44	4.1	624	3.05	32.7
Li	563	458	81.35	1.2	398	70.69	1.4	60	10.66	9.4
Dai	226	90	39.82	2.5	76	33.63	3.0	14	6.19	16.1
Zhuang	1989	710	35.70	2.8	619	31.12	3.2	91	4.58	21.9
Yao	144	42	29.17	3.4	33	22.92	4.4	9	6.25	16.0
Miao	733	199	27.15	3.7	171	23.33	4.3	28	3.82	26.2
Buyei	333	87	26.13	3.8	78	23.42	4.3	9	2.70	37.0
Han	14,233	3598	25.28	4.0	3224	22.65	4.4	374	2.63	38.1
Dong	643	154	23.95	4.2	140	21.77	4.6	14	2.18	45.9
Tujia	163	32	19.63	5.1	31	19.02	5.3	1	0.61	163.0
Sui	199	39	19.60	5.1	37	18.59	5.4	2	1.01	99.5
Bai	280	54	19.29	5.2	51	18.21	5.5	3	1.43	70.0
Yi	452	68	15.04	6.6	64	14.16	7.1	4	0.88	113.0
Va	131	15	11.45	8.7	15	11.45	8.7	—	—	—
Hani	241	10	4.15	24.1	10	4.15	24.1	—	—	—
Others	213	39	18.31	5.5	32	15.02	6.7	7	3.29	30.4
Unknown	409	112	27.38	3.7	104	25.43	3.9	8	1.96	51.1

Others included ethnicities from Hui, Lahu, Gelao, Blang, Mulao, Manchu, Lisu, Mongol, Jino, Tibetan, Maonan, Naxi, Chuangqing, De’ang, Pumi, Achang, Korean, Gin, Jingpo, San.

— indicates “not detected”

Of the 5707 carriers, ~89.07% (*n* = 5083) were positive for one disorder and 10.93% (*n* = 624) for two or more disorders (Table 2). The Li ethnicity again ranked first for multiple-disease carriers (*n* = 60, 10.66%). Individuals from the Yao and Dai ethnicities were more likely than other ethnicities to be multiple-disease carriers as well (6.25% and 6.19%, respectively). Conversely, no Hani and Va individuals carrying multiple disease-causing variants were identified with this ECS panel.

The data were stratified by the ethnicity and disease to explore the variability among ethnic groups (Table 3). It is apparent that the selected diseases are widely spread among most of the ethnic groups. Variation in thalassemia carrier frequency predominantly contributed to the variability. The Li, Dai, and Zhuang had a carrier frequency of 78.33%, 27.43%, and 22.83% for α-thalassemia and 9.59%, 11.06%, and 8.60% for β-thalassemia. Moreover, 14.32% of individuals from the Miao ethnic group were heterozygous for PAH deficiency, which was almost four times the overall frequency in the screened population (3.59%) and twice the carrier frequency among the Sui (7.04%). Finally, the Buyei group was at a greater than average risk of carrying ARPKD (1.80% vs. 0.56% overall) and the Bai of carrying DFNB1 (3.93% vs. 1.66% overall).

**Table 3:** Carrier frequencies categorized by ethnicity for each disease

Ethnicity	Number of screened	Carrier frequency (%)										
		α-thalassemia	β-thalassemia	PAH deficiency	WD	DFNB1	DFNB4/PDS	PD	ARPKD	HPABH4A	Galactosemia	Dystrophinopathies^a^
All	20,952	15.12	4.75	3.59	1.96	1.66	1.59	0.75	0.56	0.25	0.12	0.12
Han	14,233	13.21	4.26	3.30	1.91	1.86	1.74	0.74	0.53	0.33	0.15	0.16
Zhuang	1989	22.83	8.60	2.06	2.51	1.11	1.86	0.80	0.40	0.10	0.15	0.10
Miao	733	9.96	2.46	14.32	1.77	0.55	0.68	0.41	0.95	—	—	–
Dong	643	8.55	4.98	5.60	3.42	0.62	0.31	1.56	0.78	0.16	0.16	–
Li	563	78.33	9.59	0.89	1.07	0.71	0.71	0.71	0.36	—	—	–
Yi	452	4.42	1.55	4.65	1.33	1.99	0.88	0.22	0.44	0.22	—	0.42
Buyei	333	12.91	7.51	3.60	1.20	0.90	0.90	0.30	1.80	—	—	–
Bai	280	3.57	2.14	5.71	2.14	3.93	1.43	1.43	0.36	—	—	–
Hani	241	2.07	0.41	—	—	0.83	—	—	0.41	—	0.41	–
Dai	226	27.43	11.06	0.88	3.10	1.33	0.88	1.33	0.88	—	—	–
Sui	199	9.05	1.01	7.04	1.51	0.50	0.50	0.50	0.50	—	—	–
Tujia	163	4.91	3.07	4.91	2.45	3.07	0.61	1.23	—	—	—	–
Yao	144	12.50	9.72	5.56	2.78	2.08	2.08	0.69	1.39	—	—	–
Va	131	8.40	0.76	0.76	—	0.76	—	0.76	—	—	—	–
Others	213	8.45	3.76	0.94	—	0.94	4.23	1.88	—	—	—	–
Unknown	409	12.47	4.65	2.93	2.69	2.44	2.69	0.24	0.98	0.24	—	–

*PAH deficiency* Phenylalanine hydroxylase deficiency, *WD* Wilson disease, *DFNB1* GJB2-related DFNB 1 nonsyndromic hearing loss and deafness, *DFNB4* Deafness, autosomal recessive 4, with enlarged vestibular aqueduct, *PDS* Pendred syndrome, *PD* Pompe disease, *ARPKD* Autosomal recessive polycystic kidney disease

— indicates “not detected”.

^a^Carrier frequency in female

### Carrier couples

Overall, 254 carrier couples (2.42%) were heterozygous for one condition, and one carrier couple (0.01%) was heterozygous for both PAH deficiency and β-thalassemia (Table 4). Unsurprisingly, α-thalassemia was the top disease in carrier couples (*n* = 137, 1.31%), followed by β-thalassemia (*n* = 53, 0.51%) and PAH deficiency (*n* = 34, 0.32%).

**Table 4:** Carrier couples by diseases

Disease	*N*	%^a^
All	255	2.43
Carrier couples for one disease	254	2.42
α-thalassemia	137	1.31
α-thalassemia, Hb H	102	0.97
α-thalassemia, Hb Barts	31	0.30
α-thalassemia, Hb H or Hb Barts	4	0.04
β-thalassemia	53	0.51
β-thalassemia intermedia	28	0.27
β-thalassemia major	25	0.24
PAH deficiency	34	0.32
Dystrophinopathies^b^	13	0.12
Wilson disease	5	0.05
DFNB1	4	0.04
DFNB4/PDS	4	0.04
Pompe disease	2	0.02
ARPKD	2	0.02
Carrier couple for two diseases	1	0.01
β-thalassemia intermedia & Phenylalanine hydroxylase deficiency	1	0.01

*PAH deficiency* Phenylalanine hydroxylase deficiency, *DFNB1* GJB2-related DFNB 1 nonsyndromic hearing loss and deafness, *DFNB4* Deafness, autosomal recessive 4, with enlarged vestibular aqueduct, *PDS* Pendred syndrome, *ARPKD* Autosomal recessive polycystic kidney disease, *N* number of carrier couples

^a^Percentage of 10,476 couples screened

^b^Carrier frequency in female

The carrier burdens of couples by ethnicity were analyzed and groups represented by fewer than 20 couples are not listed (Supplementary Table 8). Of the forty-five Dai couples, 11.11% (*n* = 5) were carrier couples, four times greater than the average (2.43%). Of note, only 2.73% (5/183) of the Li couples were identified as carrier couples, despite a high carrier frequency in the Li ethnicity (81.35%).

## Discussion

### Selection of disorders

The aims of carrier screening are to detect carriers of genetic disorders, to enhance reproductive autonomy, and to reduce the prevalence of disability and disease in the newborn population [27]. Therefore, one of the important criteria in our selection was disease severity. All the selected diseases were either profound or severe, with the exception of DFNB1 and DFNB4/PDS, which are considered moderate disorders. These two disorders were nevertheless included due to their high prevalence in the Chinese population [28], which was corroborated by this study. In addition, screening for hearing loss provides information which can facilitate earlier intervention to improve language development in affected children [29]. Although only 11 conditions were selected for this study, we have expanded our panel to a broader range including hundreds of conditions. A more comprehensive dataset will be published at a later date.

### Comparison to published carrier frequencies

The carrier rates of thalassemia were higher in our study than published estimates for the Chinese population [30] based on the analysis of specific variants. This might be due to the adoption of targeted next-generation sequencing, which facilitated the identification of rare novel variants as well as reported variants that affect function [3, 6, 31]. Of the 376 identified deleterious or likely deleterious variants in our study, 82.98% (*n* = 312) had already been reported in the literature but 17.02% (*n* = 64) were novel, including nonsense, frameshift, deletion or duplication of exons, and splice site variants (Supplementary Table 9). The carrier frequencies of DFNB1 and DFNB4/PDS were similar to published estimates [28]. While Wilson disease has been investigated in a large Han population, the carrier frequency was not reported [32]. The remaining disorders have been subject to less investigation in the Chinese population, and study populations were either too small or limited to patients rather than including healthy individuals.

The difference in carrier frequencies compared with Western populations was striking. For example, the carrier frequency for dystrophinopathies generated in our study (1 in 806) was lower than that previously reported (1 in 433.7); likewise, 1 in 806 subjects was carrier of galactosemia, lower than the reported frequency of 1 in 100 [31]. Furthermore, the carrier rate for PAH deficiency (1 in 28) was higher compared to the Hispanic descent (1 in 163) and the southern Europeans (1 in 75) [8]. These differences may be attributed to differences in sample size, ethnic groups or variants detection range. These results also indicate that the disorders recommended for screening in Western societies might not be the correct targets in other populations.

### The need for expanding carrier screening

Our data suggest a potential need to implement ECS in China for several reasons. First, around 27.49% of individuals were carriers for at least 1 of 11 selected diseases. Even when thalassemia is removed from our analysis, 10.02% of the individuals were still positive for at least one selected disorder. It is reasonable to believe that the frequency will increase when more diseases are included in the screening panel. Second, routine prenatal/preconception clinical screening for monogenic diseases (with the exception of thalassemia) is a novel practice in Chinese health care [17–20, 33]. ECS would detect carrier couples affected by monogenic diseases other than thalassemia, which comprised 6.2‰ (65/10 476) of our study population (Table 4). Third, one couple was found to be at risk of having a fetus with both β-thalassemia intermedia and PAH deficiency simultaneously. Traditional screening strategies would require multiple tests to identify two or more disorders. Fourth, according to calculations using a published method [7], the modeled risk of fetuses being affected by any of the selected disorders was 531 per 100,000 (95% CI, 497–567 per 100,000), which translates into approximately 1 in 188 genetically affected births. This rate is comparable to the frequency of neonates affected by Down syndrome [34], for which free, government-subsidized screening is offered in routine antenatal tests in China.

We recommend that the screening strategy be universal rather than based on ethnicity. There are five reasons for this recommendation: (1) Thalassemia screening is currently recommended in southern China for all couples who are considering pregnancy or are already pregnant, regardless of their ethnicity, although the ethnic variability of thalassemia carrier frequency has been reported [18, 19]. This serves as a good example which ECS should follow. (2) We have illustrated the variability of disease carrier frequencies in the different ethnic groups (Table 3), the complexity of which makes it unrealistic to develop screening panels for each of the 56 ethnic groups. (3) Information regarding variants in the population to be tested would be needed [12], but such data are barely available in the minority groups, especially those with a smaller population. (4) Ethnic-based screening may raise the risk of discrimination or stigmatization [9]. (5) Internal geographical mobility is continuously improving in China [13], which increases the possibility of mixed marriages and hinders ethnic identification. In our data, there were over 1127 (>11%) couples of mixed ethnicity (Supplementary Table 8).

### Challenging for genetic counseling

Undoubtedly, genetic counseling is an essential part of implementing carrier screening [35]. Our study provides valuable information about causative mutation(s) and mutation frequency in different ethnic groups which will greatly help clinical genetic counselors in risk evaluation. However, challenges remain, especially in the interpretation of variants of uncertain significance (VUS) generated from NGS. For instance, while the pathogenicity of VUS is controversial or the evidence for their classification is insufficient, they cannot be neglected because VUS have been reported as disease etiologies in some patients [36]. In our data, the paternal partner in one couple had the deleterious variant NM_000277.1: c.331C>T p.(Arg111*) in the *PAH* gene, while the maternal partner had a VUS NM_000277.1: c.1144T>A p. (Phe382Ile) in the same gene. Determining the pathogenicity of p. (Phe382Ile) would be crucial for reproductive advice in this case. Furthermore, for females with an X-linked VUS detected, pathogenicity evaluation is a necessity to properly evaluate the implications for reproductive risk.

Furthermore, our data revealed that the variant spectrum of diseases in an ethnicity is uncertain and complex, which complicates genetic counseling. Specifically, around 2.73% of Li couples were found to be carrier couples (Supplementary Table 8), despite a much higher individual carrier rate of 81.35% among the Li (the α-thalassemia carrier frequency was 78.33%). This phenomenon was mainly due to the complicated variant spectrum of α-thalassemia. In the Li ethnicity, the –α^3.7^ allele and –α^4.2^ alleles account for 72.95% of mutated alleles. Since the combination of –α^3.7^ and –α^4.2^ results in α-thalassemia trait, a mild phenotype [37], couples with the two alleles were not classified as carrier couples.

### Clinical utility of ECS

Our study revealed a wide range of carrier frequencies among the different ethnic groups in Southern China, highlighting the potential value of ECS. However, we only evaluated the clinical utility of ECS in terms of carrier and risk couple frequency distribution and test feasibility and validity. A proposed framework for the assessment of clinical utility of genetic testing should include the feasibility and validity of test, diagnostic thinking, therapeutic choices, patient outcomes and societal impacts [38]. Aspects other than test feasibility and validity and carrier and risk couple frequency need to be evaluated in future research for the full assessment of the clinical utility of ECS.

In summary, we report the first expanded carrier screening in China of 10,476 couples with diverse ethnicities. The data not only revealed the carrier frequencies of 11 monogenic disorders, but also suggested the potential utility of ECS in regions with diversified ethnic backgrounds. This experience should be applicable for other countries or regions with diverse ethnic groups. We note that most of the couples screened were from the Han ethnicity (67.93%), and sample numbers of several ethnic groups were limited. However, this is consistent with the demographic characteristics of the Chinese population, in which the Han makes up the majority (91.52%) according to the Sixth National Population Census in 2010 [16]. Nevertheless, we expect further examinations of these minority groups using a larger study population. Finally, we argue for the necessity of expanded carrier screening as a promising alternative and for genetic counseling, which is indispensable in the responsible implementation of expanded carrier screening.

## Electronic supplementary material

Supplementary Figures

Supplementary Table 1

Supplementary Table 2

Supplementary Table 3

Supplementary Table 4

Supplementary Table 5

Supplementary Table 6

Supplementary Table 7

Supplementary Table 8

Supplementary Table 9
