# Supplementary material for: Pilot study of expanded carrier screening for 11 recessive diseases in China: results from 10,476 ethnically diverse couples
Source: Eur J Hum Genet. 2018 Oct 1;27(2):254–62. doi: 10.1038/s41431-018-0253-9 (PMC6336873; doi:10.1038/s41431-018-0253-9)
Supplement: Supplementary file 1 — Supplementary Figures [file 41431_2018_253_MOESM1_ESM.docx]

**
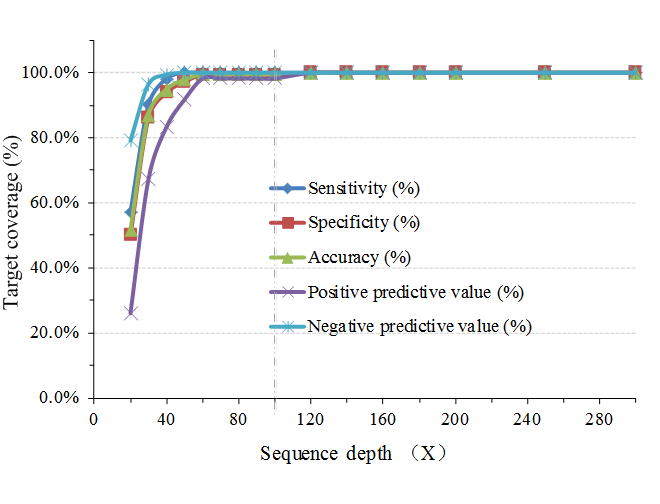
**

**Supplementary Figure 1** The variant calling performance of different sequence depths on the target regions. When the mean sequencing depth was greater than 100-fold, the sensitivity, specificity and accuracy were all greater than 99%.

**
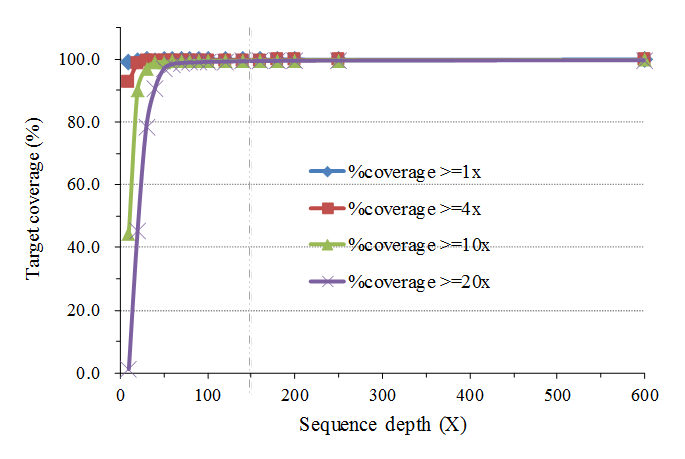
**

**Supplementary Figure 2** The coverage performance of different sequence depths on the target regions
